# Reciprocal learning and chronic care model implementation in primary care: results from a new scale of learning in primary care

**DOI:** 10.1186/1472-6963-11-44

**Published:** 2011-02-23

**Authors:** Luci K Leykum, Ray Palmer, Holly Lanham, Michelle Jordan, Reuben R McDaniel, Polly H Noël, Michael Parchman

**Affiliations:** 1 South Texas Veterans Health Care System and the Department of Medicine at the University of Texas Health Science Center at San Antonio, San Antonio, Texas, USA; 2 South Texas Veterans Health Care System and the Department of Family and Community Medicine at the University of Texas Health Science Center at San Antonio, San Antonio, Texas, USA; 3 Arizona State University, Mary Lou Fulton Teachers College, Phoenix, Arizona, USA; 4 The McCombs School of Business, the University of Texas at Austin, Austin, Texas, USA

## Abstract

**Background:**

Efforts to improve the care of patients with chronic disease in primary care settings have been mixed. Application of a complex adaptive systems framework suggests that this may be because implementation efforts often focus on education or decision support of individual providers, and not on the dynamic system as a whole. We believe that learning among clinic group members is a particularly important attribute of a primary care clinic that has not yet been well-studied in the health care literature, but may be related to the ability of primary care practices to improve the care they deliver.

To better understand learning in primary care settings by developing a scale of learning in primary care clinics based on the literature related to learning across disciplines, and to examine the association between scale responses and chronic care model implementation as measured by the Assessment of Chronic Illness Care (ACIC) scale.

**Methods:**

Development of a scale of learning in primary care setting and administration of the learning and ACIC scales to primary care clinic members as part of the baseline assessment in the ABC Intervention Study. All clinic clinicians and staff in forty small primary care clinics in South Texas participated in the survey.

**Results:**

We developed a twenty-two item learning scale, and identified a five-item subscale measuring the construct of reciprocal learning (Cronbach alpha 0.79). Reciprocal learning was significantly associated with ACIC total and sub-scale scores, even after adjustment for clustering effects.

**Conclusions:**

Reciprocal learning appears to be an important attribute of learning in primary care clinics, and its presence relates to the degree of chronic care model implementation. Interventions to improve reciprocal learning among clinic members may lead to improved care of patients with chronic disease and may be relevant to improving overall clinic performance.

## Background

Despite a well-developed evidence base regarding optimal treatments for many chronic diseases, including hypertension, type 2 diabetes, congestive heart failure, and chronic obstructive pulmonary disease, many patients seen in primary care settings do not receive these treatments [1,2]. Efforts to improve the delivery of evidence-based care have largely focused on provider knowledge [3-5] and decision support [6-11]. However, systematic reviews suggest that educational or knowledge-based interventions targeting individual providers to improve quality of care have been largely unsuccessful [12-14]. This finding suggests that we cannot depend only upon individual knowledge or decision-making capability of providers to improve care. Instead, focusing also on the larger systems in which patients receive care may lead to better results.

The chronic care model reflects this idea through its attention to not only patients and providers, but on the healthcare system itself [15]. Its focus on elements of a healthcare system that are important for chronic disease management, specifically self-management support, decision support, clinical information systems, and delivery system design, reflect the understanding that the healthcare system in which care is delivered influences chronic disease management [16]. The chronic care model, however, is not specific about the dynamics of health care systems or the evolving context in which care is delivered, nor is it specific about how these elements are implemented. Understanding these dynamics is critical to changing them, and to improving the care of patients with chronic disease [17,18].

Conceptualizing healthcare settings such as primary care as clinical microsystems gives us insight into the dynamics of clinical systems, and may make our efforts to improve chronic care delivery more effective. Clinical microsystems are the individual, functional units in which care is delivered, such as a primary care clinic, an inpatient unit, or an intensive care unit. A growing literature provides support for the application of complex adaptive system (CAS) theory to these clinical systems [19-24]. CASs are comprised of groups of individuals who learn, self-organize to complete tasks, and co-evolve with their external environment [19,22]. Additionally, they are defined by non-linearity, meaning that inputs and outputs may not be proportional or even necessarily predictable. In a CAS, the inter-dependencies among the agents are as important if not more important than the characteristics of the agents in understanding system outcomes.

These attributes of CAS suggest that the ability to learn is critically important. Learning is a social, shared process through which individuals incorporate new information in ways that lead them to change their mental models and adapt. The ability to learn can help people deal with an uncertain and changing environment more effectively. There is evidence to support the importance of learning in clinical microsystems: in operating room teams where learning occurs more effectively throughout the group, new techniques are more quickly adapted [25]; when learning occurs in nursing homes, patients receive better care [26].

Despite this insight, the phenomenon of learning in clinical microsystems is not well understood. We sought to better understand the ways in which learning occurs in primary care settings and to relate learning to primary care clinic performance. To accomplish this, we first developed a scale designed to measure attributes of learning based on the literature related to learning in the organizational and educational psychology fields. We report the development of this learning scale and the factor analysis of the scale items. To understand the association between learning and clinic performance, we then analyzed the association between learning scale scores and degree of chronic care model implementation, as measured by the Assessment of Chronic Illness Care (ACIC) scale [27]. We chose the ACIC scale because we believe chronic disease management is a critical function of primary care clinics, and because ACIC scores have been linked to patient outcomes [28,29]. We hypothesized that provider and staff ratings of learning would be associated with their assessment of the extent to which the chronic care model had been implemented in their clinics.

## Methods

### Development of a learning scale

We convened a multidisciplinary team with expertise in improving provider behavior and organizational change. In 2006-2007, we conducted a targeted search focused on pulling together a diverse set of papers that discussed learning in terms of a social activity that is inherent in organizations, teams, and individuals. We focused on the organizational learning and educational psychology literatures, beginning with key papers that operationalize learning in organizations [30-33], learning in teams [34,35], and learning by individuals [36-38]. We expanded our review by working backwards and forwards, examining works referenced by those authors and references of those authors in subsequent publications. This literature was synthesized by three team members into a summary of themes associated with learning, shown in Additional file 1[30-47]. With the assistance of the fourth team member, items were developed to explore the presence of these learning themes in primary care settings. We believed that learning would be embedded in the following types of clinic member actions: asking questions beyond the presenting issue, sharing knowledge about a patient or a disease, staff and patient education, learning as things happen in the clinic, and learning from unexpected events or mistakes. We also believed that learning would occur through conversation and reflection. These understandings formed the basis of the questions about learning.

We created a new scale consisting of twenty-two items reflecting the learning themes identified in our literature review. The scale instructs respondents to indicate their level of agreement with each statement using a 5-point Likert scale. Responses for each item are scored from one (strongly agree) to five (strongly disagree). Scale items were pilot tested in three Veterans Affairs (VA) primary care clinics and two VA contract clinics in South Texas and administered to one hundred and one staff and providers across those five clinics, including front desk staff, medical assistants, nurses, and physicians. Cronbach's alpha for the learning questions based on this sample was 0.814, indicating good internal consistency. Based on feedback and questions from participants in the pilot, the wording of specific items was refined. This refinement consisted primarily of changing negatively worded items to positive ones, and using the word "I" consistently instead of "we." The final list of items is shown in Additional file 2.

### Administration of the learning survey

The ABC study is a cluster randomized controlled trial testing the effectiveness of a practice facilitation intervention to improve the processes of care and outcomes for diabetic patients in forty primary care clinics in South Texas. As part of this study, a baseline survey that included the learning scale items was administered to all clinicians and office staff of these primary care clinics prior to the start of the intervention by the research team. Here we report on the results of the baseline cross-sectional survey.

The primary care clinics included in the ABC study are generally small, autonomous, physician-owned clinics with four or fewer primary care providers. Thirty of the clinics have only one physician, and of these thirty, eleven had one or more non-physician providers (either physician assistant or nurse practitioner). Ten clinics had two to four physicians and of those, five had at least one physician assistant or nurse practitioner. No clinics had other types of providers such as nutritionists or counselors.

### Chronic care model implementation assessment

The extent to which each clinic provides optimal care for patients with chronic illnesses was measured with the Assessment of Chronic Illness Care scale (ACIC) [27]. The ACIC is a twenty-five item questionnaire that asks health care providers to rate the degree of support for each of the six elements of the Chronic Care Model (CCM) in their health care system: delivery system redesign, patient self-management support, decision support, information support, community linkages, and health system support. Response choices for each item range from zero to eleven, with eleven representing optimal chronic care support. In addition to a total score reflecting overall CCM implementation, the ACIC score can be split into six sub-scales that reflect each of the elements contained in the model. Version 3.5 of the ACIC was used in this study, and in addition to the 6 sub-scales, also includes items that address how well a practice integrates the CCM elements [48]. Preliminary data indicate the ACIC is responsive to changes chronic care delivery and correlates well with other measures of productivity and system improvements [27]. Prior research by members of this team also suggest that ACIC scores are associated with clinical outcomes such as A1c control and ten-year risk of a cardiovascular event. That is, patients who attend clinics with higher ACIC scores have lower A1c values and lower risk [28,29].

We included the ACIC in the baseline survey completed by all clinic members in the forty clinics enrolled in the ABC study.

### Factor analysis of the learning survey

We performed a principal components factor analysis of the learning scale [49]. Eigenvalues over 1, scree plot inspection, and determination of simple structure across items were used to identify potential factors. Cronbach coefficient alpha scores in the range of 0.7 were used to identify those factors with the greatest degree of internal validity.

### Association between learning survey and ACIC

We calculated Pearson correlation coefficients between subscales identified in the factor analysis, total ACIC scores, and ACIC sub-component scores related to each element of the CCM.

## Results

Two-hundred and ninety-six respondents from 40 clinics completed the survey during the period from October 2007 to May 2010. Fifteen percent of these were physicians, and 6% non-physician providers. The remainder of the respondents were other clinic staff members, such as front desk staff or medical assistants. Characteristics of the clinics surveyed are shown in Table 1. Medicare is the government-sponsored heathcare program for persons over age 65 in the United States, and reflects the proportion of geriatric patients in each practice. The number of managed-care contracts is a reflection of the number of insurers with which each practice is contracted.

**Table 1:** Characteristics of surveyed clinics

Clinic characteristic	Mean	Median	Range
Number of providers(MD,DO,PA,NP)	2.6	2	1 to 7

Number of staff	5.7	5	2 to 12

% clinics with EHR's	64%		

Number of active patients in practice	4267	3350	1000 to 12000

Number of patient visits/week	136.3	150	175 to 315

Number of managed care contracts	14.1	5	0 to 70

% Medicare patients	34	40	0 to 80

% practices with a patient tracking or registry system	5%		

% practices experiencing a financial loss in the last three years	24%		

% practices hosting students or residents	52%		

% practices holding regular business meetings	76%		

Principal components factor analysis revealed three factors with Cronbach coefficient alpha scores of 0.82, 0.57, and 0.68. Factor loading ranged from 0.44 to 0.77. The factors with scores of 0.57 and 0.68 were eliminated based on being below our acceptability threshold, and items not being conceptually similar. We examined the eight items in the factor with a score of 0.82. Based on the conceptual content included in these items and their factor loading, we concluded that five of the eight items were capturing an idea of learning as a shared, back-and-forth process between clinic members. We called this concept "reciprocal learning" to reflect what we believed was the notion of reciprocal interdependency - an interdependency in which the output of a system is produced by the collaboration of all contributing entities, and in which these entities are dependent on each other to produce the optimal system-level output [50]. The specific items in the reciprocal learning factor are shown in Table 2. The Cronbach alpha for the five items was 0.79. The mean score for each item across clinics was 3.83 (SD = 0.72), with a range from 1.4 to 5. The specific scores for each individual item in the reciprocal learning factor are shown in Additional file 3.

**Table 2:** Items in the reciprocal learning subscale identified by factor analysis

Survey Item
I am frequently taught new things by other people in this clinic

I learn a lot about how to do my job by talking with the people in the clinic

When we have a problem in this clinic, we tend to examine it carefully so that we can come to an understanding of the problem and why it occurred

In this clinic, we frequently learn about new things together as a group

I learn how to do things in this clinic by sharing knowledge with team members

The mean, median, and range in ACIC scores and component scores across clinic are shown in Table 3. These scores indicate that there was a broad range in the extent to which practices had implemented the CCM elements. Inspection of normalized residual plots and skewness statistics reveal that all the variables in our analysis conformed to normal distributions, as do the close correspondence between the mean and median of each variable in the table.

**Table 3:** Mean, median and range in ACIC scores across clinics

ACIC component	Mean scores	Median scores	Range across clinics
Total ACIC score	204.0 (74.8)	204.0	0 - 374

Community linkages	16.1 (9.3)	16.0	0-33

Self-management support	24.2 (9.8)	24.0	0-44

Decision support	23.5 (9.9)	23.5	0-44

Delivery system design	38.6 (13.4)	38.0	0-66

Clinical information systems	28.6 (13.1)	27.0	0-55

Health system support	35.8 (14.9)	35.0	0-66

Integration of elements	40.1 (14.4)	42.0	0-66

Table 4 shows the Pearson correlations between learning scale scores and ACIC total and component scores. Correlation between the reciprocal learning and the ACIC score and subscales ranged from 0.28 to 0.46. We adjusted this analysis to account for the clustering effect of consistency of responses within clinics to reduce the potential bias that could result from clustering. The intraclass correlations (ICC) of the variables ranged from .10 to .22 suggesting that respondents within clinic tend to answer in a similar manner therefore affecting standard error estimates. MLWin software [51] was used to obtain unbiased associations. Adjusted correlations are also shown in Table 4.

**Table 4:** Association between reciprocal learning sub-scale and ACIC total and component scores

ACIC Component	Correlation coefficient	p-value	Correlation coefficient adjusted for clustering effect	p-value
Total ACIC score	.44	<.0001	.38	<.0001

Community linkages	.28	<.0001	.26	<.0001

Self-management support	.40	<.0001	.35	<.0001

Decision support	.39	<.0001	.33	<.0001

Delivery system design	.45	<.0001	.39	<.0001

Clinical information system	.38	<.0001	.25	<.001

Health system support	.43	<.0001	.38	<.001

Integration of elements	.45	<.0001	.42	<.0001

## Discussion

We sought to better understand learning in primary care clinics, and the relationship between learning and clinic performance as measured by the degree to which the CCM was present in primary care clinics. To accomplish this, we first developed and administered a twenty-two item learning scale that reflected 6 learning themes described in the organizational and educational psychology literature. We then performed a factor analysis to examine which items most closely clustered together in the scale responses. We used the resulting factors to better understand which aspects of learning were most relevant within primary care settings. This analysis identified a subset of five items that reflect a learning process that occurs between people where each learns from sharing with the other, and in which the learning acquired from one person becomes the foundation for further learning by others in a building, iterative process. Because of the mutual and iterative nature of this process, we believe it reflects the concept of reciprocal learning [50]. We found a wide range of responses across clinics to the items on the five-item reciprocal learning scale, indicating that responses to the items on this scale can be used to discriminate between the clinics.

To better understand the role of learning in primary care settings, we wanted to understand the possible association between learning and clinic performance. Because we view the care of chronic illness and the presence of the chronic care model elements to be critical aspects of primary care delivery, we used ACIC scores as a measure of primary care clinic performance and tested the association between reciprocal learning and the ACIC. Reciprocal learning was significantly associated with ACIC scores, suggesting that this type of learning may be particularly important for successful chronic care model implementation.

This conceptualization of learning moves beyond the idea of one person learning from another to that of people learning together, building on each other's understandings. These findings echo studies from operating room teams and nursing home caregivers that demonstrated the importance of each individual contributing to care in a shared way [25,34]. The literature related to learning in healthcare settings is limited, and our results should be considered a first step in the development of the concept of reciprocal learning in these settings. However, because learning is a social activity that is dependent on relationships and the ability of clinic members to have the opportunity to speak to each other, studies on relationships, conversation, and reflection [52-56] in healthcare settings complement our findings.

Our design of developing a scale to understand learning has several limitations. First, we developed our scale based on descriptions of learning in non-healthcare disciplines. While physicians and researchers with knowledge of primary care settings applied the concepts in ways that would be meaningful to healthcare providers in the development of the survey items, we may have missed aspects of learning important to healthcare settings that were not part of other disciplines. Second, using a scale administered at a single point in time may not be optimal for describing a dynamic and evolutionary process such as learning. Despite this, our results do discriminate between clinics and point to what we believe is an important concept of reciprocal learning. Finally, our findings are limited in that we only included forty small primary care clinics in South Texas. These results may not translate as easily to larger primary care practices or more integrated group settings, or in other geographic areas.

Despite these limitations, our findings are an important step forward in understanding the role of learning in primary care clinics. This understanding may be particularly important in light of efforts to implement patient-centered medical home (PCMH) care models [57,58] in United States primary care settings. The purpose of the PCMH is to provide patient-centered care in which all clinic members are engaged and responsible in the care of all patients. Reciprocal learning may be an important way to improve engagement of clinic members and their ability to learn from each other to improve patient care. Improved care of patients with chronic disease is an important part of the PCMH model, and the CCM elements are shared with those of the PCMH. Successfully implementing these models of care is not a simple or static process. It requires not only attention to multiple aspects of the system in which care is delivered, but also an emphasis on patients' support system, and their ability to manage their diseases. To accomplish this requires the active and proactive engagement of staff and providers to be alert and open to new ways of doing things, to understand the impact of the way they do things on others, and to learn not only from, but with each other and respond to the needs of their patients with chronic illnesses [55]. This may explain why reciprocal learning is associated with the degree to which the chronic care model was present in each clinic.

Interpretation of our results underscores the idea that the kinds of learning required in clinical microsystems are more sophisticated than typically acknowledged. Learning is an interdependent process that occurs between and among all members of the clinic. Managing learning as an interdependent process will likely be difficult, but our findings suggest that it will be necessary to improving the care delivered to patients with chronic disease.

## Conclusions

We describe the construct of reciprocal learning in primary care clinics, an activity through which clinic members learn from each other in an iterative, building process. Reciprocal learning appears to be an important attribute of learning in primary care clinics, as its presence relates to the degree of chronic care model implementation. Interventions to improve reciprocal learning among clinic members may lead to improved care of patients with chronic disease and may be relevant to improving overall clinic performance. Reciprocal learning may also be important for clinics' ability to move to more patient-centered models of care.

## Competing interests

The authors declare that they have no competing interests.

## Authors' contributions

MJ carried out the literature review of learning with assistance from HL. MJ, HL, RRM led the development of the learning scale. MP conceived and carried out the ABC study in collaboration with PHN. RP performed the statistical analysis. LL drafted the manuscript. All authors were involved in review and interpretation of the reported results. All authors read and approved the final manuscript.

## Pre-publication history

The pre-publication history for this paper can be accessed here:

http://www.biomedcentral.com/1472-6963/11/44/prepub

## Supplementary Material

Additional file 1 **Themes or activities related to learning identified by literature search**. This file lists the six themes related to learning identified in the literature search and provides references and examples for each. Click here for file

Additional file 2 **Learning Scale items**. This file lists the twenty-two items in the final learning scale administered in this study. Click here for file

Additional file 3 **Description of scores on each item in the reciprocal learning scale**. This file lists the items in reciprocal learning scale and lists the minimum, maximum, and mean scores and standard deviation for each item. Click here for file
