# Supplementary material for: Reciprocal learning and chronic care model implementation in primary care: results from a new scale of learning in primary care
Source: BMC Health Serv Res. 2011 Feb 23;11:44. doi: 10.1186/1472-6963-11-44 (PMC3050698; doi:10.1186/1472-6963-11-44)
Supplement: Additional file 1 — Themes or activities related to learning identified by literature search. This file lists the six themes related to learning identified in the literature search and provides references and examples for each. [file 1472-6963-11-44-S1.DOC]

**Additional file 1: Themes or activities related to learning identified by literature search**

| **Theme** | **Examples** |
| --- | --- |
| Reflection [31] | - Learning that occurs when people take action and look to see what happened as a result of that action. - Learning that occurs when people make adjustments to action based on what they see by thinking and/or exchanging ideas with others about previous action. - Learning that occurs when people share with and listen to each other what they think happened and what they think should be done differently in the future. |
| Proactive [34] | - Learning that occurs at times when there does not seem to be a particular issue that is pressing/urgent at that moment. - Learning that occurs through the seeking out of opportunities to discover something new that is thought to be relevant to the work of the system – even if at that moment the learner cannot directly tie the target of the learning to a particular task (learning that is anticipatory of future needs). |
| Continuous [32,33,36,39-43] | - Learning that occurs through perpetual seeking or scanning for opportunities to discover new things and/or ideas (a mindset/stance toward learning and the world). |
| Shared [32,35,36,38-42,44,45] | - Learning that occurs when people teach and learn new things from each other. - Learning that occurs when people discover new things together. |
| Mindful [37] | - Learning that occurs through the continuous creation of new categories. - Learning that occurs through openness to new information. - Learning that occurs through the awareness of more than one perspective: the recognition of the existence of more than ‘one right way’ to do things well. |
| Sources of Learning [30,46,47] | - Learning that occurs because of the use of multiple resources. - Learning that occurs because of the use of diverse resources. |
