# Supplementary material for: Reciprocal learning and chronic care model implementation in primary care: results from a new scale of learning in primary care
Source: BMC Health Serv Res. 2011 Feb 23;11:44. doi: 10.1186/1472-6963-11-44 (PMC3050698; doi:10.1186/1472-6963-11-44)
Supplement: Additional file 2 — Learning Scale items. This file lists the twenty-two items in the final learning scale administered in this study. [file 1472-6963-11-44-S2.DOC]

**Additional file 2: Learning Scale items**

| *This survey is aimed at understanding the role of learning in this clinic. When we talk about learning, we mean all of the different kinds of activities that happen in this clinic related to learning. For example, we are interested in things such as how people in this clinic learn about the patients it serves, or how people in this clinic keep up with new information about health care delivery, or how people in this clinic learn ways to improve in their jobs.* |
| --- |
| **Question** |
| 1. At work, I have time to think about how I am doing at my job in this clinic. 2. I often get together with people in this clinic to talk about our work. 3. I often consider changing the work I do in this clinic because of things I have learned. 4. When I learn in this clinic, what I learn is focused on specific problems. 5. Even when things are going well in this clinic, I think about changing the way things are done around here. 6. When we have a problem in this clinic, we tend to examine it carefully so that we can come to an understanding of the problem and why it occurred. 7. I am encouraged to experiment with new ways of doing things. 8. In this clinic, we look for one right way to do things. 9. I need to learn all the time in order to be successful at my job. 10. I frequently teach other people in this clinic new things. 11. I am frequently taught new things by other people in this clinic. 12. In this clinic, we frequently learn about new things together as a group. 13. I learn how to do things in this clinic by sharing knowledge with team members. 14. I would be more effective in my work if people in this clinic would leave me alone and let me do my job. 15. I have a lot of flexibility in how I do my work. 16. In this clinic, we are encouraged to follow practice guidelines. 17. In this clinic we focus on trying to tailor treatments to fit the specific needs of each patient. 18. My learning in this clinic often focuses on matters that lead me to question what I already know. 19. I have many resources that I can use to increase my knowledge about how I do my job. 20. I go outside of the clinic to learn new things to help me with my job. 21. I learn a lot about how to do my job by talking with the people in the clinic. 22. People in this clinic ask other people in this clinic how they do things. |
