# Supplementary material for: Reciprocal learning and chronic care model implementation in primary care: results from a new scale of learning in primary care
Source: BMC Health Serv Res. 2011 Feb 23;11:44. doi: 10.1186/1472-6963-11-44 (PMC3050698; doi:10.1186/1472-6963-11-44)
Supplement: Additional file 3 — Description of scores on each item in the reciprocal learning scale. This file lists the items in reciprocal learning scale and lists the minimum, maximum, and mean scores and standard deviation for each item. [file 1472-6963-11-44-S3.DOC]

**Additional file 3: Description of scores on each item in the reciprocal learning scale**

| **Learning scale item** | **Minimum score** | **Maximum score** | **Mean Score** | **Standard Deviation** |
| --- | --- | --- | --- | --- |
| 6 | 1 | 5 | 3.9 | 1.02 |
| 11 | 1 | 5 | 3.7 | 0.93 |
| 12 | 1 | 5 | 3.5 | 1.01 |
| 13 | 1 | 5 | 4.3 | 0.77 |
| 21 | 1 | 5 | 3.9 | 0.99 |
